# Adipose triglyceride lipase decrement affects skeletal muscle homeostasis during aging through FAs-PPARα-PGC-1α antioxidant response

**DOI:** 10.18632/oncotarget.8552

**Published:** 2016-04-01

**Authors:** Katia Aquilano, Sara Baldelli, Livia La Barbera, Daniele Lettieri Barbato, Giuseppe Tatulli, Maria Rosa Ciriolo

**Affiliations:** ^1^ Department of Biology, University of Rome ‘Tor Vergata’, Rome, Italy; ^2^ Università Telematica San Raffaele Roma, Rome, Italy; ^3^ IRCCS San Raffaele ‘La Pisana’, Rome, Italy

**Keywords:** antioxidants, lipid metabolism, myoblasts, oxidative stress, inflammation, Gerotarget

## Abstract

During aging skeletal muscle shows an accumulation of oxidative damage as well as intramyocellular lipid droplets (IMLDs). However, although the impact of these modifications on muscle tissue physiology is well established, the direct effectors critical for their occurrence are poorly understood. Here we show that during aging the main lipase of triacylglycerols, ATGL, significantly declines in gastrocnemius and its downregulation in C2C12 myoblast leads to the accumulation of lipid droplets. Indeed, we observed an increase of oxidative damage to proteins in terms of carbonylation, S-nitrosylation and ubiquitination that is dependent on a defective antioxidant cell response mediated by ATGL-PPARα-PGC-1α. Overall our findings describe a pivotal role for ATGL in the antioxidant/anti-inflammatory response of muscle cells highlighting this lipase as a therapeutic target for fighting the progressive decline in skeletal muscle mass and strength.

## INTRODUCTION

With the term “sarcopenia” is usually indicated a progressive decline in skeletal muscle mass and strength, a process that occurs physiologically during aging and is responsible for the weakness and compromised locomotion of the elderly [1]. Several factors were indicated to be responsible for the onset of such condition, but no efficient interventions were established for its avoidance. Among them the most stated are: reduction of physical activity, decreased number and increased irregularity of muscle units, lowered anabolic hormones and enhanced cytokine activity [1].

At the molecular level, it has been evidenced that an unbalanced reactive oxygen species (ROS) burst is a hallmark in the processes underlying aging/sarcopenia of skeletal muscle, with increased amount of oxidative damage to lipids, DNA and proteins [2]. However, which of the common molecular redox-sensitive pathways are critical for the oxidative damage remains uncertain. Considerable data pointed out that aged-skeletal muscle becomes unable to activate the antioxidant response pathways to cope with ROS deriving from muscle contraction activity, indicating that redox transcription factors are indispensable for maintaining the integrity of muscles [3, 4].

Recently, we have delineated an antioxidant cellular response aimed at counteracting oxidative/nitrosative stress, a circumstance occurring in several age-related diseases and healthy aging upon shortage of canonical antioxidants, such as glutathione. Indeed, under glutathione withdrawal, the peroxisome proliferator-activated receptor gamma, coactivator 1 alpha (PGC-1α) is up-regulated in various tissues including skeletal muscle. PGC-1α enforces the transcription of the redox-sensitive nuclear factor (erythroid-derived 2)-like2 (NFE2L2), which in turn promotes the expression of the antioxidant enzymes such as manganese superoxide dismutase (SOD2) and γ-glutamylcysteine ligase (γ-GCL) [5]. Even though the molecular mechanism through which PGC-1α exerts this effect was not identified, the PGC-1α/NFE2L2 axis is of interest in skeletal muscle function where PGC-1α plays fundamental roles, also related to mitochondrial homeostasis.

Reduction in basal rates of mitochondrial oxidative phosphorylation is another common age-related process concurring to muscle derangements that could be responsible for the increased intramyocellular lipid droplets (IMLDs) accumulation. In particular, immunohistochemical studies carried out on muscle biopsies from young (22 years) and old (73 years) individuals demonstrated that older adults have larger IMLDs, fewer mitochondria and a lower proportion of IMLDs in contact with mitochondria [6]. These modifications are important aspects of the skeletal muscle aging process, which leads to an unbalance of lipid and oxidative metabolism and mitochondrial dysfunctions [7].

In order to be funneled towards the mitochondrial oxidative degradation, fatty acids (FAs) need to be firstly conjugated in triacylglycerols (TAGs) and stored in lipid droplets (LDs), which represent the reservoir of “aerobic” energy. LDs are dynamic organelles responding to energy and lipid homeostasis in a very tight-regulated manner. Therefore, their number and size are dependent on the activity of hydrolytic lipases, which are able to completely disassemble TAGs. Three different types of lipases, often hormonally-regulated, are known, among which the adipose triglyceride lipase (ATGL) catalyzes the first step of the TAGs lipolysis cascade, *i.e.* the hydrolytic cleavage of TAGs into FAs and diacylglycerols (DAGs). ATGL is predominantly expressed in adipose tissue and present, to a lesser extent, in testis, cardiac and skeletal muscle [8]. In particular, ATGL is exclusively expressed in type I (oxidative) muscle fibers, where it possibly plays an indispensable role in FAs metabolism [9]. In fact, ATGL deletion in mice yielded a phenotype with increased whole body fat mass and neutral lipids accumulating in adipose and non-adipose tissues [10].

FAs liberated by ATGL, besides being used by mitochondria for energy production, have been implicated in lipid signaling mediated by the family of peroxisome proliferator activated receptors (PPARs) [11]. In particular, PPARα-activation induces a negative transcriptional regulation of nuclear transcription factor-kappa B (NF-kB) and activating protein-1 (AP-1) [12], while it stimulates the antioxidant response through increased expression of superoxide dismutase and catalase [13]. Moreover, we demonstrated that, during aging, adipocytes exhibit impaired activation of ATGL and PPARα-mediated lipid signaling pathway that results in the up-regulation of pro-inflammatory cytokines, such as TNFα and IL-6, highlighting a fundamental role of ATGL in counteracting age-related inflammation [14, 15].

On the basis of this knowledge, we hypothesized an involvement of ATGL and PPARα-mediated lipid signaling in skeletal muscle and a possible impairment of such processes during aging. To test this hypothesis, we assessed the expression of established PGC-1α target genes in relation to the aforementioned antioxidant response in skeletal muscle during aging. We showed that a progressive decline of ATGL expression characterizes muscle aging and was accompanied by defects in the antioxidant response. These events were recapitulated in young ATGL-KO mice, indicating that ATGL is essential in orchestrating the FAs-PPARα-PGC-1α antioxidant/anti-inflammatory response.

## RESULTS

### Oxidative/nitrosative stress and inflammation correlate with ATGL down-regulation and fibers atrophy in skeletal muscle of old mice

The progression of aging is well known to result in reduction of mitochondrial content in skeletal muscle and whole-body muscle mass (sarcopenia) [16]. Next to this, an accumulation of IMLDs has been observed mostly in human type I fibers and in rhesus monkeys during aging [6, 17]. Similar defects in lipid accumulation have been observed in humans suffering from neutral lipid storage disease with myopathy (NLSDM), a rare disorder caused by different mutations in the gene coding for ATGL [18, 19]. Indeed, these patients accumulate large amounts of TAGs in skeletal muscle that confers muscle weakness and skeletal muscle myopathy [20].

**Figure 1:**
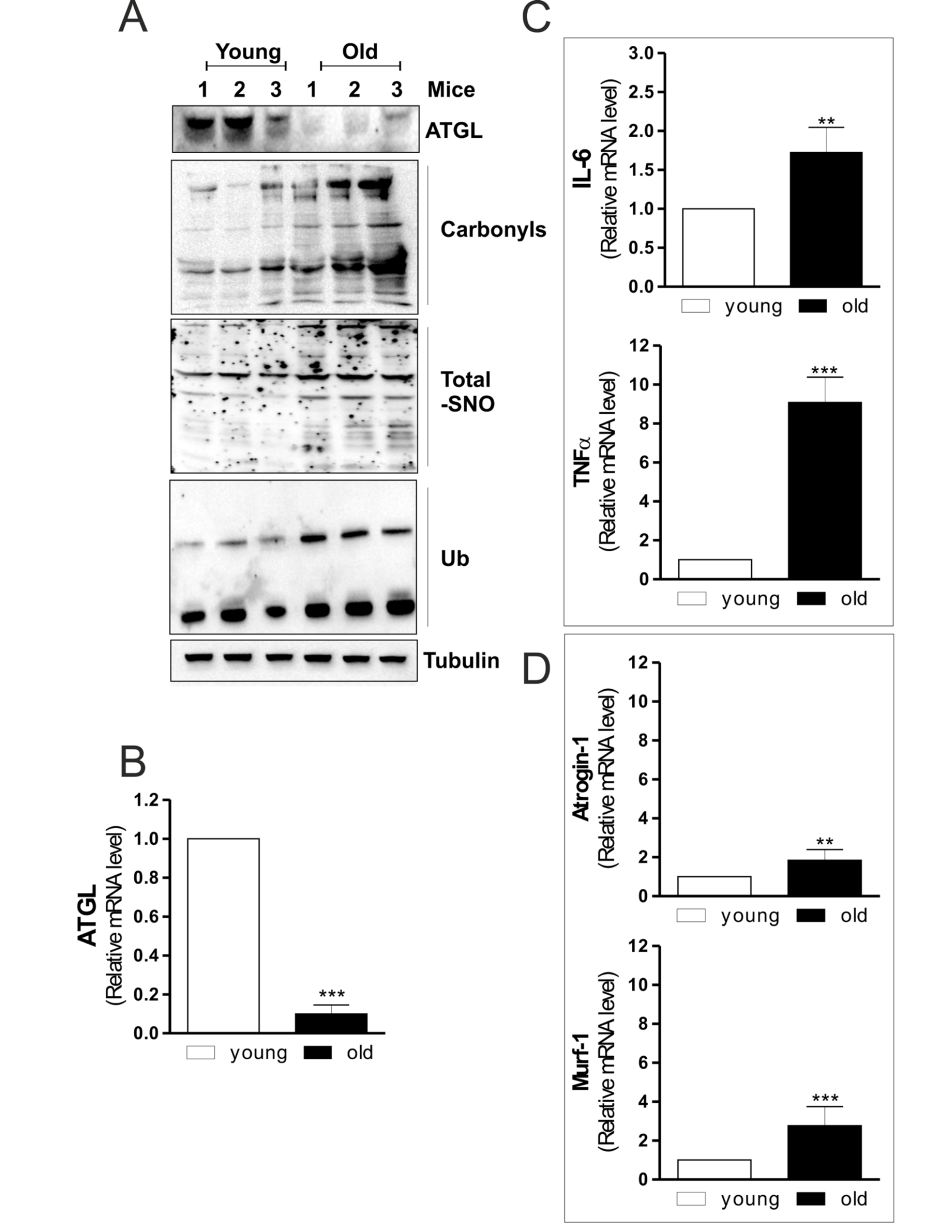
ATGL is decreased in skeletal muscle of old mice **A.** Skeletal muscle of three young and three old mice was homogenized and 20 μg of total proteins were subjected to Western blot analysis of ATGL. Twenty μg of total proteins were derivatized with DNP and carbonylation was detected by Western blot with DNP antibody (upper panel). Five-hundred μg of total proteins were subjected to S-NO derivatization with biotin. After Western blot, biotin adducts were identified by incubating nitrocellulose membrane with HRP-conjugate streptavidin (middle panel). Twenty μg of total proteins were used to detect ubiquitinated proteins by immunoblot with an anti-ubiquitin monoclonal antibody (bottom panel). Tubulin was used as the loading control. All the immunoblots reported are from one experiment representative of four that gave similar results. **B.**, **C.**, **D.** Total RNA was isolated from skeletal muscle of three young and three old mice and relative mRNA levels of ATGL, IL-6, TNFα, Atrogin-1 and Murf-1 were analyzed by RT-qPCR. Data are expressed as means ± S.D. (*n* = 3, **p < 0.001, ***p < 0.0001 *vs.* young mice).

Given that the IMLDs metabolism is tightly dependent upon the activity of intracellular lipases, we hypothesised that specific lipases managing TAGs catabolism could be affected also in skeletal muscle of old mice. In particular, we looked at ATGL, which is exclusively expressed in type I fibers of skeletal muscle. These fibers are classified as slow-twitch according to the mode of metabolism (aerobic, phosphorylation) and are characterized by high TAGs storage compared to type II fibers (anaerobic, glycolysis). Figure 1A displays that old mice have ATGL protein level significantly decreased compared to young mice. Moreover, RT-qPCR analysis shows a dramatic reduction of ATGL mRNA (Figure 1B), indicating an affected lipolytic cascade in myofibers.

Aging correlates also with increased oxidative damage in skeletal muscle that contributes to loss of tissue homeostasis [4]. Impairment of redox balance has been demonstrated to induce oxidative modifications of proteins, including carbonylation and ubiquitination [16, 21]. Thus, we measured the extent of protein oxidation and ubiquitination in the total protein lysates. As shown in Figure 1A, a raise in total carbonylated and ubiquitinated proteins is observed in old mice, which exhibit also a higher extent of protein S-nitrosylation (Figure 1A), confirming an imbalance of both oxidative and nitrosative conditions during aging.

Inflammation is another important condition in age-related skeletal muscle degeneration [22]. Moreover, a modulatory action of ATGL on tissue inflammation has been recently reported in cardiac muscle of ATGL-KO mice [23]. In particular, the steatotic heart of these animals shows a prominent up-regulation of inflammatory markers [23]. Accordingly, we found that skeletal muscle of old mice displayed an increase of the pro-inflammatory cytokines such as IL-6 and TNF-α (Figure 1C). From these results it could be suggested that skeletal muscle of old mice is prone to conditions that lead to degeneration of myofibers. Then we analyzed the level of fiber atrophy by measuring the content of Atrogin-1 and Murf-1, E3 ubiquitin ligases that are important regulators of ubiquitin-mediated protein degradation in skeletal muscle [24]. Old mice displayed a significant increase of Atrogin-1 and Murf-1 mRNA levels (Figure 1D), confirming the occurrence of skeletal muscle degenerative program during aging.

Overall the results so far obtained, while in part confirmatory of the detrimental age-associated processes, outline a prominent decrement of ATGL in old mice. Moreover, it is worth noting that ATGL decrement could be the upstream event responsible for both oxidative/inflammatory condition and IMLDs accumulation. To this aim we next deeply dissected the molecular mechanisms triggered by ATGL downregulation in C2C12 myoblasts.

### ATGL downregulation leads to accumulation of inflammatory markers in C2C12 myoblasts and myofibers

**Figure 2:**
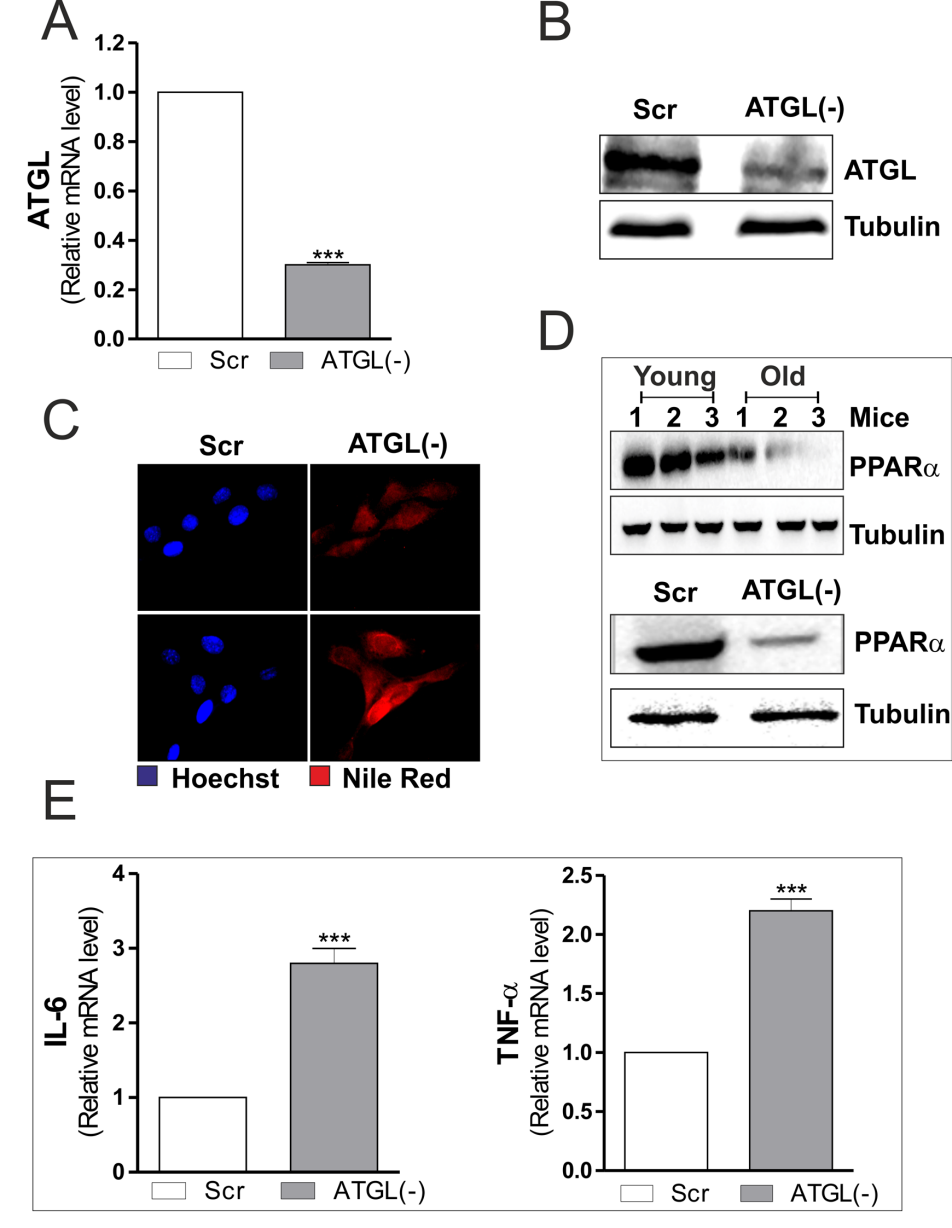
ATGL down-regulation is associated with increased pro-inflammatory markers in C2C12 myoblasts **A.** C2C12 cells were transfected with scramble (scr) or ATGL siRNA (ATGL(−)). Total RNA was isolated, and relative mRNA level of ATGL was analyzed by RT-qPCR. Data are expressed as means ± S.D. (*n* = 4, ***p < 0.0001 *vs*. scr cells). **B.** Twenty μg of total proteins were subjected to Western blot analysis of ATGL. Tubulin was used as loading control. **C.** Determination of TAGs content by Nile Red staining in scr and ATGL(−) myoblasts. **D.** Skeletal muscle of three young and three old mice was homogenized and 20 μg of total proteins were subjected to Western blot analysis of PPARα (upper panel). scr and ATGL(−) cells were lysed and 20 μg of total proteins were subjected to Western blot analysis of PPARα (bottom panel). Tubulin was used as loading control. All the immunoblots reported are from one experiment representative of three that gave similar results. **E.** Total RNA was isolated, and relative mRNA levels of IL-6 (left panel) and TNFα (right panel) were analyzed by RT-qPCR. Data are expressed as means ± S.D. (*n* = 4, ***p < 0.0001 *vs*. scr cells).

To dissect the link between ATGL decrease and inflammation we down-regulated its expression by means of siRNA. C2C12 myoblasts were transfected with a siRNA against ATGL (ATGL(−)) or with a scramble siRNA (scr). Downregulation of ATGL was successfully achieved as both its mRNA and protein content were significantly decreased (Figure 2A and 2B). Moreover, ATGL down-regulation led to the accumulation of LDs (Figure 2C), demonstrating the effective impairment of lipid catabolism in ATGL(−) cells.

ATGL-released FAs control the expression and activity of PPARα that is considered a powerful repressor of inflammatory genes [12] and an activator of antioxidant enzymes (i.e. superoxide dismutase, catalase) [25, 26]. Moreover, we previously demonstrated that upon ATGL down-regulation FAs/PPARα signalling was impaired in visceral adipose tissue and adipocytes during aging [14]. The same experimental conditions also determine an induction of inflammation [14, 15]. Thus, we investigated the level of PPARα and pro-inflammatory genes both in ATGL-down-regulating C2C12 myoblasts and in skeletal muscle during aging. The analysis of PPARα protein level revealed that its expression was significantly dampened in old mice and in ATGL(−) myoblasts (Figure 2D). In line with the anti-inflammatory role of ATGL/FAs/PPARα pathway, ATGL down-regulation in C2C12 myoblasts led to an enhanced expression of pro-inflammatory cytokines as evidenced by increased mRNA levels of IL-6 and TNF-α (Figure 2E). Therefore, on the basis of these data we can suggest that the rise of pro-inflammatory cytokine production, observed during aging, could be at least in part due to the failure of ATGL-FAs-PPARα-mediated signalling pathways.

### ATGL downregulation causes an alteration of antioxidants in C2C12 myoblasts and in myofibers

**Figure 3:**
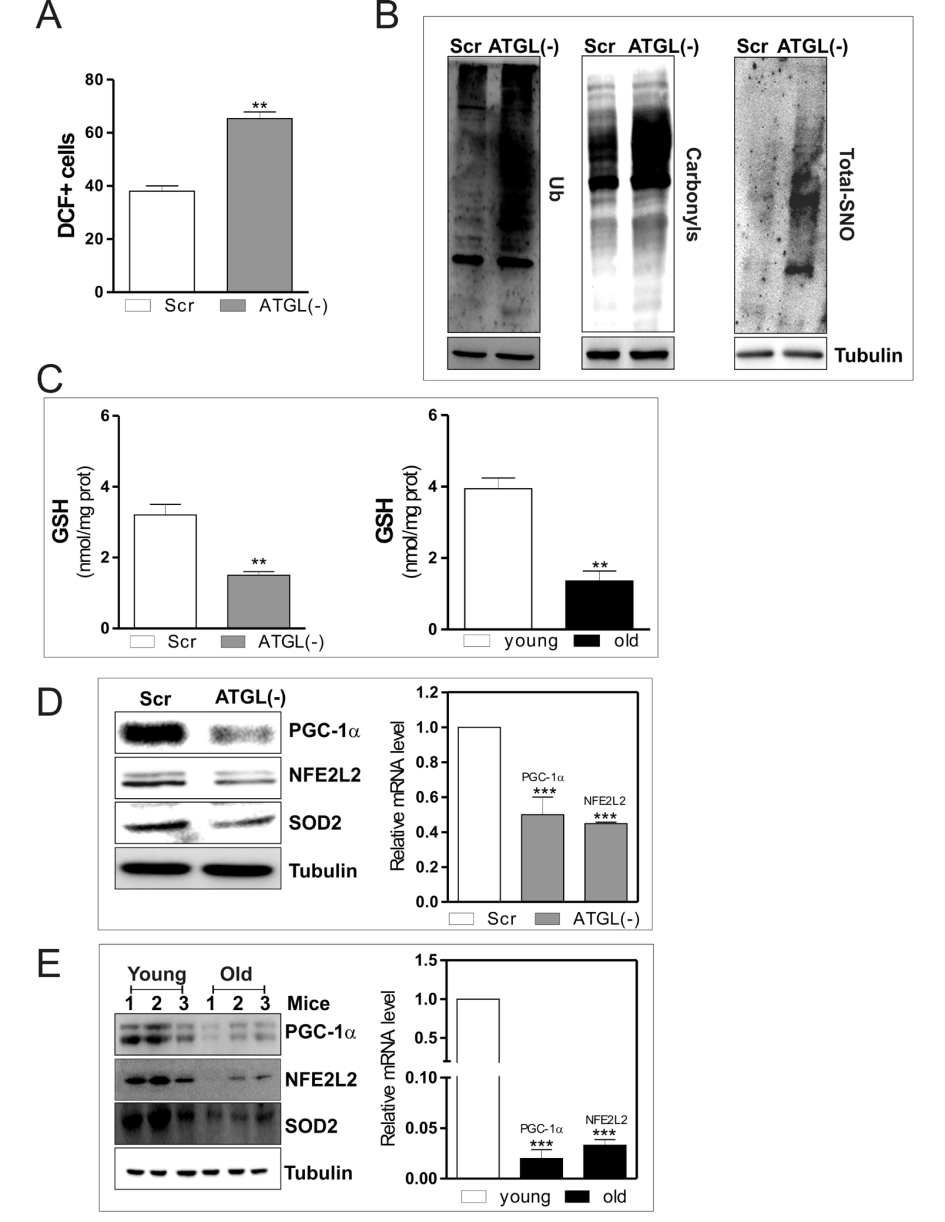
ATGL down-regulation induces an antioxidant imbalance in skeletal muscle C2C12 cells were transfected with scramble (scr) or ATGL siRNA (ATGL(−)). **A.** Cells were assayed for ROS/ONOO- production by cytofluorimetric analysis after DCF-DA staining. ROS/ONOO- level was reported as percentage of DCF-positive cells and expressed as means ± S.D. (*n* = 4, **p < 0.001 *vs*. scr cells). **B.** Twenty μg of total proteins were used to detect ubiquitinated proteins by immunoblot with an anti-ubiquitin monoclonal antibody (left panel). Twenty μg of total proteins were derivatized with DNP and carbonylation was detected by Western blot with DNP antibody (middle panel). Total proteins extracts (500 μg) were subjected to S-NO derivatization with biotin. After Western blot, biotin adducts were identified by incubating nitrocellulose membrane with HRP-conjugate streptavidin (right panel). Tubulin was used as the loading control. **C.** GSH content of scr, ATGL(−) cells (left panel) and of three young and three old mice (right panel) was assayed by HPLC. Data are expressed as nmoles of GSH/mg of proteins and reported as means ± S.D. (*n* = 3, **p < 0.001 *vs*. scr cells or young mice). **D.**. scr and ATGL(−) cells were lysed and 20 μg of total proteins were subjected to Western blot analysis of PGC-1α, NFE2L2 and SOD2 (left panel). Tubulin was used as loading control. Total RNA was isolated from scr and ATGL(−) cells (right panel) and relative mRNA levels of PGC-1α and NFE2L2 were analyzed by RT-qPCR. Data are expressed as means ± S.D. (*n* = 4, ***p < 0.0001 *vs*. scr cells). **E.** Skeletal muscle of three young and three old mice was homogenized and 20 μg of total proteins were subjected to Western blot analysis of PGC-1α, NFE2L2 and SOD2 (left panel). Tubulin was used as loading control. All the immunoblots reported are from one experiment representative of four that gave similar results. Total RNA was isolated from skeletal muscle of three young and three old mice and relative mRNA levels of PGC-1α and NFE2L2 were analyzed by RT-qPCR. Data are expressed as means ± S.D. (*n* = 4, ***p < 0.0001 *vs*. young mice).

Next we analyzed role of ATGL in modulating oxidative metabolism and antioxidant defence in skeletal muscle. We first monitored the production of ROS/ONOO^.−^ in ATGL(−) cells. Figure 3A shows an augmented percentage of DCF^+^ cells as consequence of an increased ROS/ONOO^.−^ flux in ATGL(−) with respect to scr cells. ROS/ONOO^.−^ imbalance was accompanied by increased levels of oxidized proteins in terms of ubiquitinated, carbonylated and S-nitrosylated (Figure 3B). These results indicated that ATGL deficiency might be causative of the oxidative stress observed in skeletal muscle during aging. Moreover, we measured the levels of glutathione (GSH), the main intracellular non-enzymatic antioxidant that safeguards protein integrity by impending ROS- and NO-mediated damage [27]. Figure 3C shows that GSH was significantly decreased in ATGL(−) cells as well as in skeletal muscle of old mice.

We previously demonstrated that the disruption of the redox buffer controlled by GSH leads to PGC-1α up-regulation that is directed to potentiate the antioxidant defence through NFE2L2-mediated expression of SOD2 and γ-GCL [5]. PGC-1α is a master controller of cell metabolism and represents a down-stream target of the ATGL-governed lipid signalling [28]. Therefore, we evaluated whether the ATGL decrease results in the impairment of PGC-1α-mediated antioxidant response. As expected a reduction of protein and mRNA levels of PGC-1α were observed in ATGL(−) cells (Figure 3D) as well as in old mice (Figure 3E). Thus, we moved to prove that reduced ATGL-PPARα-PGC-1α signaling pathway leads to defective antioxidant response during aging.

**Figure 4:**
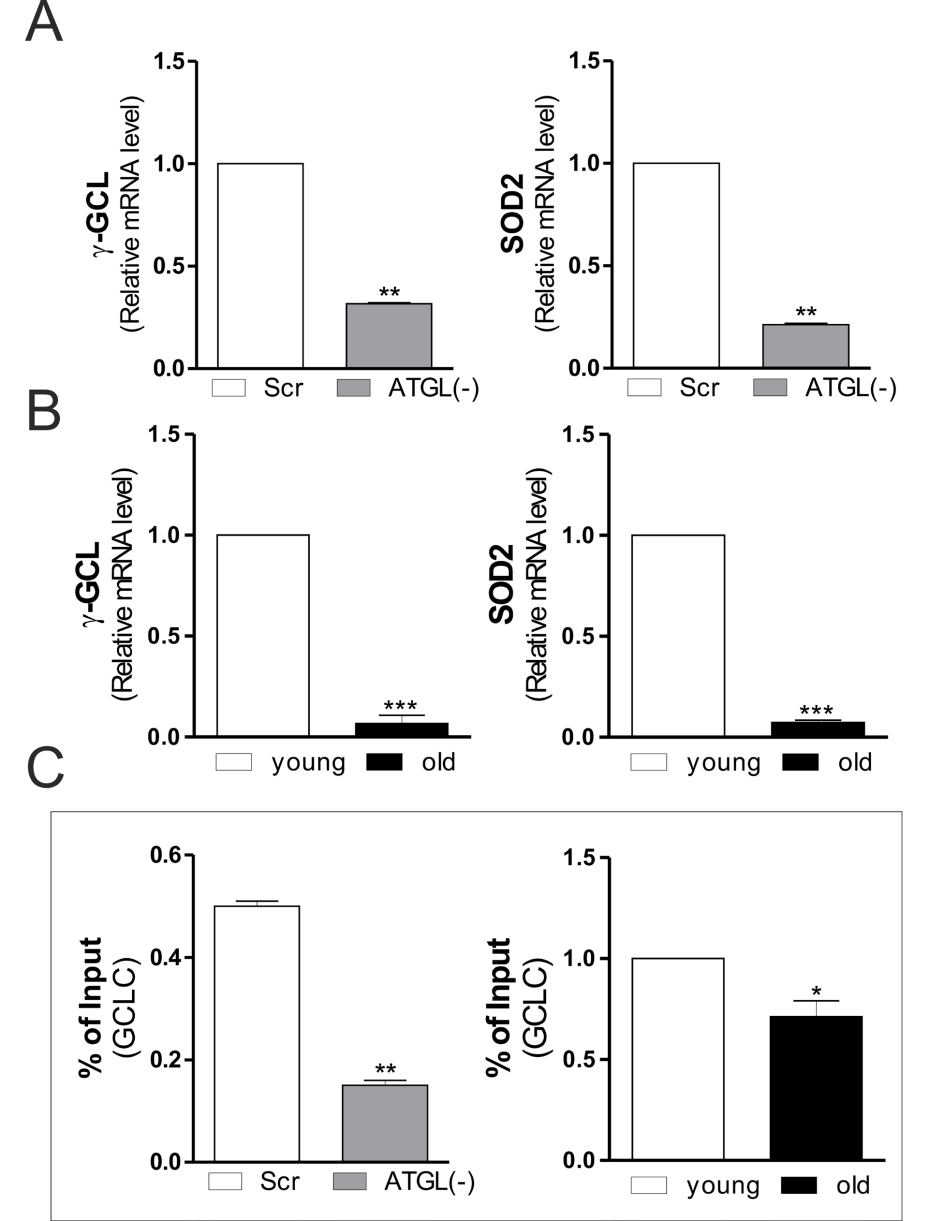
Changes in SOD2 and γ-GCL gene expression upon ATGL decline in skeletal muscle **A.**, **B.** Total RNA was isolated from scr, ATGL(−) cells (upper panels) and skeletal muscle of three young and three old mice (bottom panels), and relative mRNA levels of γ-GCL and SOD2 were analyzed by RT-qPCR. Data are expressed as means ± S.D. (*n* = 3, **p < 0.001; ***p < 0.0001 *vs*. scr cells or young mice). **C.** ChIP assay was carried out on cross-linked nuclei from scr, ATGL(−) cells and skeletal muscle of three young and three old mice by using NFE2L2 antibody followed by qPCR analysis of ARE sequence on the GCLC promoter. Data are expressed as means ± S.D. (*n* = 3, **p < 0.001;*p < 0.01 *vs*. scr cells or young mice).

The analysis of the protein and mRNA levels of NFE2L2 demonstrated that it was significantly reduced in ATGL(−) cells (Figure 3D) and in old mice (Figure 3E). We then evaluated whether ATGL-downregulation could inhibit PGC-1α/NFE2L2-governed antioxidant response. The protein (Figure 3D and 3E) and mRNA (Figure 4A and 4B) levels of SOD2 and γ-GCL were significantly decreased in ATGL(−) cells and in old mice, confirming an impairment of ATGL-PPARα-PGC-1α-mediated antioxidant response. To verify whether the binding activity of NFE2L2 complex was modulated by ATGL deficiency, NFE2L2 association with promoter of the mouse GCLC gene, coding for the catalytic subunit of γ-GCL, was assayed. ChIP analysis indicated that NFE2L2 binding activity was decreased in the occupancy of NFE2L2 responsive element in the GCLC promoter upon AGTL down-regulation as well as in old mice (Figure 4C).

In summary, these results highlight the role of ATGL in orchestrating the antioxidant response in skeletal muscle during aging. In particular, ATGL by providing FAs, is able to activate the PPARα-PGC-1α complex, which in turn induces the expression of NFE2L2-mediated antioxidant genes (i.e. SOD2 and γ-GCL), preventing oxidative stress.

### Skeletal muscle of young ATGL-KO mice resemble that of aged mice

**Figure 5:**
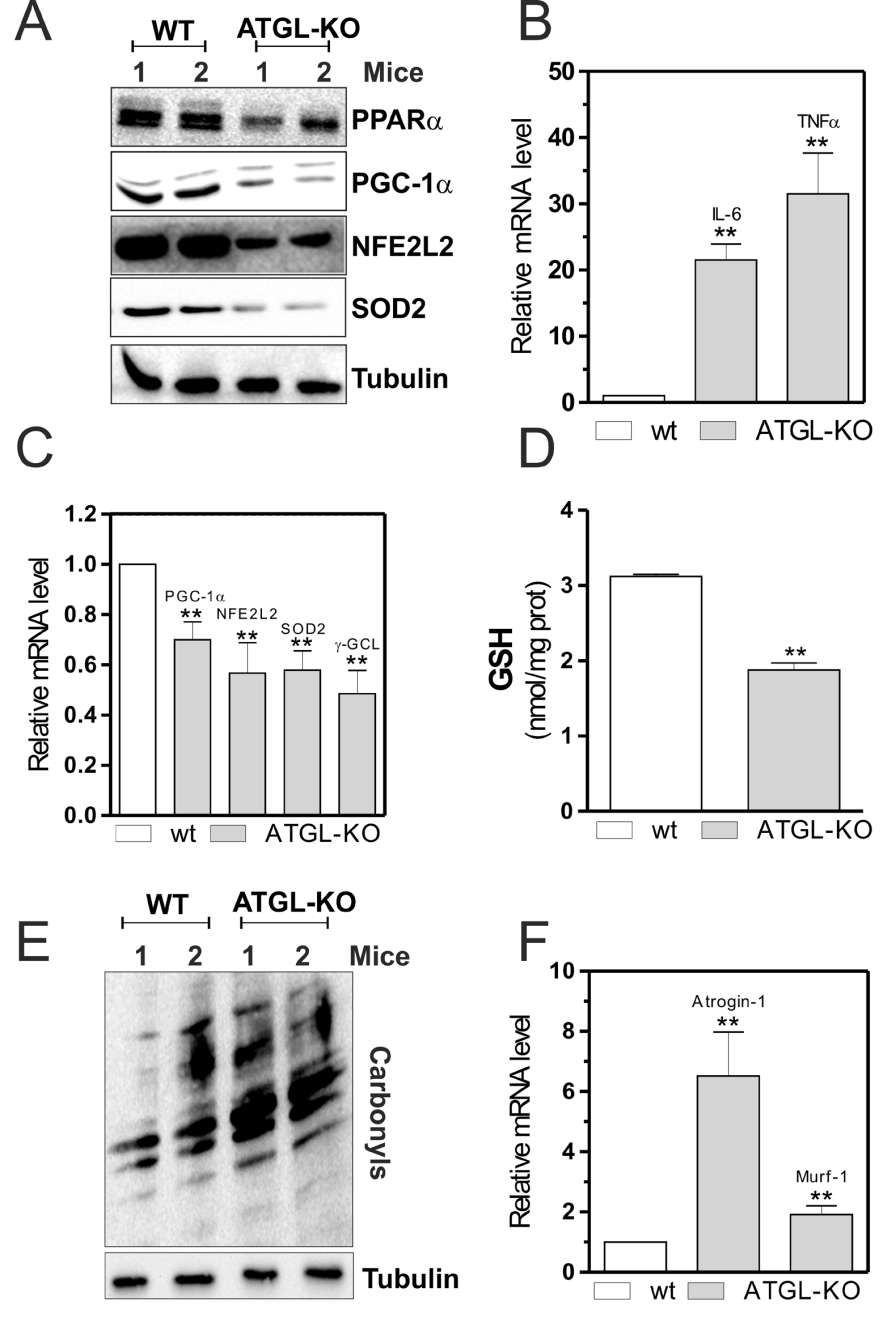
PPARα-PGC-1α-mediated antioxidant pathway, oxidative stress and skeletal muscle degeneration in ATGL-KO mice **A.** Skeletal muscle of two wild type (WT) and two ATGL-KO mice was homogenized and 20 μg of total proteins were subjected to Western blot analysis of PPARα, PGC-1α, NFE2L2 and SOD2. Tubulin was used as loading control. **B.**, **C.** Total RNA was isolated from skeletal muscle of two WT and two ATGL-KO mice, and relative mRNA levels of IL-6, TNFα, PGC-1α, NFE2L2, SOD2 and -GCL were analyzed by RT-qPCR. Data are expressed as means ± S.D. (*n* = 3, **p < 0.001 *vs*. WT mice). **D.** GSH content of WT and ATGL-KO mice was assayed by HPLC. Data are expressed as nmoles of GSH/mg of proteins and reported as means ± S.D. (*n* = 3, **p < 0.001 *vs*. WT mice). **E.** Twenty μg of total proteins were derivatized with DNP and carbonylation was detected by Western blot with DNP antibody. Tubulin was used as loading control. All the immunoblots reported are from one experiment representative of five that gave similar results. **F.** Total RNA was isolated from skeletal muscle of WT and ATGL-KO mice, and relative mRNA levels of Atrogin-1 and Murf-1 were analyzed by RT-qPCR. Data are expressed as means ± S.D. (*n* = 3, **p < 0.001 *vs*. WT mice).

To confirm the essential role of the lipolytic activity of ATGL in maintaining skeletal muscle redox homeostasis and functionality, we analyzed some of the molecular factors previously observed in the murine model of ATGL-KO. Although the ATGL-KO mice used were young (six weeks), their skeletal muscles showed the same characteristics of old wild-type (WT) mice. In particular, ATGL-KO mice displayed a decrease of PPARα (Figure 5A) that was associated with a powerful induction of pro-inflammatory cytokines (IL-6 and TNF-α) in a similar way to that observed in old mice and in ATGL(−) cells (Figure 5B). Moreover, skeletal muscle of ATGL-KO mice displayed decreased protein (Figure 5A) and mRNA (Figure 5C) levels of PGC-1α, NFE2L2, SOD2 and γ-GCL. This phenomenon was associated with decreased level of GSH (Figure 5D) and accumulation of carbonylated proteins (Figure 5E). Next, we evaluated the levels of atrophy markers also in ATGL-KO young mice and found a significant increase of their expression (Figure 5F) respect to WT mice as it happens in aged mice, confirming that ATGL deficiency could influence the integrity of skeletal muscle.

All these results demonstrate that the generation of FAs by ATGL provides ligands for functional signaling by the PPARα-PGC-1α complex, which through NFE2L2 is able to activate the antioxidant response and maintain skeletal muscle function and homeostasis. The progressive decrease of ATGL in aged skeletal muscle causes a lipolytic defect that results in massive lipid accumulation, drastic reduction of PPAR-α–regulated gene expression, defective antioxidant response and severe skeletal muscle dysfunction (atrophy).

## DISCUSSION

Several data and hypotheses present in literature stated for multifactorial origin of old-associated sarcopenia. However, a well-characterized hallmark of such condition is the presence of IMLDs. In this work, we show how the catabolism of cellular TAGs has a key role in maintaining antioxidant response and functionality of aged skeletal muscle. In particular, this process requires the hydrolytic activity of ATGL. In fact, besides its role in adipose tissue lipolysis, ATGL plays an important role in TAGs metabolism and storage of skeletal muscle [29]. In particular, it has been demonstrated that ATGL is exclusively expressed in type I oxidative muscle fibers, which are characterized by high TAGs content compared to type II fibers, suggesting that this enzyme plays an important role in intramuscular FAs handling [9].

Aged skeletal muscle undergoes a process of structural and functional remodelling leading to loss of muscle mass and force (sarcopenia) [30]. This phenomenon is accompanied by an accumulation of oxidative damage that may contribute to loss of tissue homeostasis and atrophy [31]. The cause of this increased oxidative damage is unclear, but it might be assumed that during aging the ability of skeletal muscle to respond to ROS imbalance is very attenuated. Moreover, previous investigations indicated that intermuscular fat increases with advancing age [32], and magnetic resonance spectroscopy [33] and ultrastructural [6] studies showed that elderly subjects have greater IMLDs accumulated in type I fibers. Haemmerle and colleagues have shown that progressive lipids accumulation and myocardial fibrosis in heart of ATGL-KO mice was accompanied by left ventricular hypertrophy and impaired left ventricular systolic function [10], suggesting that the correct enzymatic activity of ATGL is a limiting condition for the cardiac muscle functionality and homeostasis. Thus, the identification of potential target/signalling pathway could help in alleviating aging-dependent oxidative damage and atrophy.

Our findings delineate an important concept in aged skeletal muscle physiology, underlying that ATGL-mediated lipolysis is essential for the maintenance of PPARα-PGC-1α-mediated antioxidant response and for skeletal muscle function and homeostasis. In particular, we initially found a progressive decrease of ATGL expression in skeletal muscle of C57BL/6 old mice. This decline causes lipolytic defects that result in: i) lipid accumulation, ii) drastic increase of oxidatively damaged proteins, and iii) severe decrease of antioxidant response. A similar phenotype is observed also in gastrocnemius/soleus of young ATGL-KO mice as well as in ATGL-deprived C2C12 myoblasts, indicating that this enzyme is responsible for TAG turnover and FAs-PPARα-antioxidant target genes expression also in skeletal muscle. In fact, ATGL-released FAs play an important role in the regulation of oxidative metabolism. Actually, the hydrolytic activity of ATGL generates lipolytic products, which can behave as ligand or ligand precursors (e.g. FAs, acyl-CoA and FAs-derived compounds) for functional signaling by the PPARα–PGC-1α complex of mitochondrial biogenesis, OXPHOS complexes and antioxidant response [11].

In our work, we substantiated that progressive decline or absence of ATGL leads to an impairment of lipid signaling and downstream antioxidant defense, determining an increase of oxidative/nitrosative stress. We have previously demonstrated that during the progressive decline of GSH, occurring with normal aging, the activation of PGC-1α/NFE2L2 pathway stimulates a sort of adaptive response that buffers the potential harmful effect of ROS/RNS, resulting in mild oxidative stress and protection against cell death [5, 34]. In line with this, the lack of ATGL-mediated FAs/PPARα signaling results in decreased expression of PGC-1α. Consistent with its established roles in skeletal muscle, mitochondrial biogenesis, substrate oxidation and prevention of oxidative stress [35], low skeletal muscle PGC-1α levels may contribute to accumulation of ROS/RNS and impaired antioxidant response [36]. Here we show that, these events are accompanied by an accumulation of damaged proteins that are eliminated by ubiquitin-proteasome system. Moreover, the loss of proteins and organelles likely result in the degeneration and atrophy of aged and ATGL-KO myofibers. In fact, we show an up-regulation of the muscle-specific atrophy-related ubiquitin ligases Atrogin-1/MAFbx and Murf-1 that accelerates protein turnover *via* the ubiquitin-proteasome system.

The important role of PGC-1α in mechanism controlling muscle mass loss has been recently reported. The maintenance of high levels of PGC-1α during catabolic conditions spares muscle mass upon aging and sarcopenia [37-39]. The positive action on muscle mass of this cofactor is due to the inhibition of ubiquitin-proteasome degradation. PGC-1α reduces protein breakdown by inhibiting the transcriptional activity of FoxO3 and NF-κB [40]. Thus, in our work the decrease of this co-activator could unlock the functionality of proteolytic systems by activating the action of the pro-atrophy transcription factors.

In this context, preliminary results from us pointed out that ATGL decrement is effective also at late stages of myoblasts differentiation and it could be dampened by treatment with MG132, a proteasome inhibitor, implying a prominent role of this degradation pathway in controlling ATGL turnover (personal communication). This is in line with data present in literature indicating that ATGL protein expression was strongly stabilized by the same proteasome inhibitor [41].

FAs can regulate cell metabolism and inflammation. The lipid-sensing PPARs transcription factors link lipid metabolism to inflammatory processes. In particular, PPARα signaling is an important key player not only in energy metabolism, but also in inflammatory response [42]. In fact, it has been demonstrated that PPARα functions like a repressor of inflammation [43]. An induction of FAs/PPARα-mediated inflammation has been observed during ATGL deficiency in visceral adipose tissue and adipocytes [14, 15]. Moreover, PPARα-KO mice show a prolonged inflammatory response [44]. The anti-inflammatory role of PPARα in skeletal muscle is strongly supported by the analysis of PPARα levels in old mice and young ATGL-KO mice, which harbored a significant reduction of PPARα levels concomitant to the production of inflammatory mediators. Our findings suggest that the aged skeletal muscle is a smoldering inflammatory state driven by cytokines, protein catabolism and oxidative stress, which may interfere with muscle contraction and induce ultimately to sarcopenia.

Actually, individuals affected by NLSDM showed altered lipid accumulation in tissues including skeletal muscle, with variable clinical symptoms, except myopathy [20]. Most NLSDM mutations lead to a truncated form of ATGL that, though lacking the C-terminal region (where the LD-binding domain is located), is not affected in lipolytic activity.

Here we give proof of the importance of the FAs generation by ATGL in skeletal muscle during aging. We propose that the progressive decline of ATGL in aged skeletal muscle could be the *primum movens* for oxidative/inflammatory conditions. In fact, the massive IMLDs accumulation, accompanied by a reduction of their oxidation, could be responsible for inflammation and oxidative damage that, at the end, would lead to skeletal muscle atrophy. These findings reveal how ATGL, through the promotion of lipid signaling, functions as anti-oxidant/anti-inflammatory-protein *via* the induction of FAs/PPARα-PGC-1α-mediated pathway in young skeletal muscle and indicate that the alteration of this signaling axis is a fundamental event in age-associated skeletal muscle dysfunction.

## MATERIALS AND METHODS

### Animals

We conducted all mouse experimentations in accordance with accepted standard of humane animal care and with the approval by relevant national (Ministry of Welfare) and local (Institutional Animal Care and Use Committee, Tor Vergata University, Rome, Italy) committees. C57BL/6 mice were purchased from Harlan Laboratories Srl (Urbino, Italy). Male and female ATGL-KO mice, and the corresponding WT mice, were generously provided by Prof. R Zechner and Dr. G Heammerle (Institute of Molecular Biosciences, University of Graz, Graz, Austria). ATGL-KO mice were generated by targeted homologous recombination and were backcrossed on a C57BL/6 genetic background [10]. 12- and 80-weeks-old mice were considered as young and old mice, respectively. Mice were fed *ad libitum* with standard pellet diet and free access to water. Before sacrifice mice were subjected to fasting for seven hours. Mice were killed by cervical dislocation, gastrocnemius/soleus muscle was explanted immediately, frozen on dry ice and stored −80¼C.

### Cell cultures

The murine skeletal muscle C2C12 cells were obtained from the European Collection of Cell Cultures (Salisbury, UK). C2C12 myoblasts were cultured in growth medium composed of Dulbecco's Modified Eagle's Medium (DMEM) supplemented with 10% fetal bovine serum, 100 U/ml penicillin/streptomycin and 2 mM glutamine (Lonza Sales, Basel, Switzerland) and maintained at 37°C in an atmosphere of 5% CO_2_ in air.

### Transfection

24 h after plating, C2C12 myoblasts were transfected with a siRNA duplex directed against the following mouse ATGL (SASI_Mm01_00082035) target sequence (ATGL(−) cells). Transfection with a scramble siRNA duplex (scr), with no homology to other mouse mRNA, was used as control. Cells were transfected by electroporation as described previously [45] and transfection efficiency of siRNA was evaluated by co-transfecting siRNAs with nonspecific rhodamine-conjugated oligonucleotides. Only experiments that gave transfection efficiency of 80% were considered. Twenty-four hours after transfection (day 0), differentiation was induced.

### Western blot analysis

Cell pellets were resuspended in RIPA buffer (50 mM Tris-HCl, pH 8.0, 150 mM NaCl, 12 mM deoxycholic acid, 0.5% Nonidet P-40 and protease inhibitors). Protein samples were used for SDS-PAGE followed by Western blotting as previously described [46]. Nitrocellulose membranes were stained with primary antibodies against Tubulin (1:1000), PGC-1α (1:500), SOD2 (1:2000), NFE2L2 (1:1000), ATGL (1:1000), Ub (1:1000) and PPARα (1:1000). Afterward, the membranes were incubated with the appropriate horseradish peroxidase-conjugated secondary antibody, and immunoreactive bands were detected by a Fluorchem Imaging System upon staining with ECL Select Western Blotting Detection Reagent (GE Healthcare, Pittsburgh, PA, USA; RPN2235). Immunoblots reported in the figures are representative of at least four experiments that gave similar results. Tubulin was used as the loading control.

### Determination of protein carbonylation

Carbonylated proteins were detected using the OxyBlot Kit (Millipore, S7150) as previously described [47]. Briefly, 20 μg of total proteins were reacted with 2,4 dinitrophenylhydrazine (DNP) for 15 min at 25¼C. Samples were resolved on 10% SDS-polyacrylamide gels and DNP-derivatized proteins were identified by Western blot analysis using an anti-DNP antibody and an appropriate horseradish peroxidase-conjugated secondary antibody.

### RT-pPCR analysis

Total RNA was extracted using TRI Reagent (Sigma-Aldrich) and used for retro-transcription as previously described [48]. Three micrograms of RNA was used for retro-transcription with M-MLV (Promega). qPCR was performed in triplicates by using validated qPCR primers (BLAST), Ex TAq qPCR Premix and the Real-Time PCR LightCycler II (Roche Diagnostics, Indianapolis, IN, USA). mRNA levels were normalized to RPL mRNA, and the relative mRNA levels were determined by using the 2−^ΔΔCt^ method. The primer sequences are listed in Table 1.

**Table 1:** List of primers used for RT-qPCR and ChIP analysis

Genes	Sequences
ATGL FW ATGL RV	5′-CAACGCCACTCACATCTAC-3′ 5′-GGACACCTCAATAATGTTG-3′
Atrogin-1 FW Atrogin-1 RV	5′-GCGACCTTCCCCAACGCCTG-3′ 5′-GGCGACCGGGACAAGAGTGG-3′
*gclc* FW *gclc* RV	5′-GCGAGGTTTCTGCTTAGTCA-3′ 5′-ACAATGACTAAGCAGAAACCTCG-3′
g-GCL FW g-GCL RV	5′-CGCACAGCGAGGAGCTTCGG-3′ 5′-CTCCACTGCATGGGACATGGTGC-3′
IL-6 FW IL-6 RV	5′-CTCTGCAAGAGACTTCCATCCA-3′ 5′-GACAGGTCTGTTGGGAGTGG-3′
Murf-1 FW Murf-1 RV	5′-AGGGGCTACCTTCCTCTAAGTG-3′ 5′-TCTTCCCCAGCTGGCAGCCC-3′
NFE2L2 FW NFE2L2 RV	5′-TCCGCCAGCTACTCCCAGGTTG-3′ 5′-TGGGCCTGATGAGGGGCAGTG-3′
PGC-1α FW PGC-1α RV	5′-ACTGCAGGCCTAACTCCTCCCAC-3′ 5′-CCCTCTTGGTTGGCGGTGGC-3′
PPARα FW PPARα RV	5′-TGCAGGTCATCAAGAAGAC-3′ 5′-TGTGCAAATCCCTGCTCTCC-3′
RPL FW RPL RV	5′-GTACGACCACCACCTTCCGGC-3′ 5′-ATGGCGGAGGGGCAGGTTCTG-3′
SOD2 FW SOD2 RV	5′-GTGTCTGTGGGAGTCCAAGG-3′ 5′-AGCGGAATAAGGCCTGTTGT-3′
TNFα FW TNFα RV	5′-ATGGCCTCCCTCTCATCAGT-3′ 5′-CTTGGTGGTTTGCTACGACG-3′

### Evaluation of ROS content

ROS were detected by cytofluorimetric analysis after incubation for 30 min at 37¼C with 50 μM H2DCF-DA as previously reported [49]. The fluorescence intensity of 10.000 cells from each samples were analyzed by FACScalibur instrument (Beckton-Dickinson, San Jose, CA, USA). Data were analyzed using the WinMDI 2.8 software (Scripps Research Institute, La Jolla, CA, USA).

### Chromatin immunoprecipitation assay (ChIP)

ChIP was carried out as previously described [5]. Briefly, after cross-linking of nuclei extracted from C2C12 cells were fragmented by ultrasonication using 4×15 pulse (output 10%, duty 30%). Samples were pre-cleared with pre-adsorbed salmon sperm Protein G agarose beads (1 h, 4°C), and overnight immunoprecipitation using anti-NFE2L2 or control IgG antibody was carried out. After de-cross-linking (1% sodium dodecyl sulfate at 65°C for 3 h), qPCR was used to quantify the promoter binding with 30 cycles total (95°C, 1 s; 60°C, 30 s; 72°C, 60 s). Results are expressed as percentage of Input (1%) values. The primers used are reported in Table 1.

### Biotin switch assay

Biotin switch assay was performed as previously described [50]. Briefly, proteins were subjected to S-NO derivatization by incubation in the presence of ascorbate, which reduces S-NO groups. The same sample incubated in the presence of biotin without ascorbate was used as negative control. After protein separation by non-reducing SDS-PAGE and Western blot, biotinylated proteins were detected by incubation of nitrocellulose membrane with HRP-conjugated streptavidin (1:1000).

Protein concentration was determined by the method of Lowry *et al.* [51].

### Determination of GSH

Intracellular GSH level was measured by HPLC as previously described [52].

### Lipid assays

TAGs were detected by incubation with 0.25 mg/ml fluorescent Nile Red and staining was microscopically analysed.

### Statistical analysis

The results are presented as means ± S.D. Statistical evaluation was conducted by ANOVA, followed by the post-Student-Newman-Keuls test. Differences were considered to be significant at *p* < 0.05.
